# Difference in Perfusion Parameters Between Gastric Cancer and Gastric Stromal Tumors: Evaluation With Oral Contrast Plus Contrast-Enhanced Ultrasonography

**DOI:** 10.3389/fonc.2020.00532

**Published:** 2020-05-05

**Authors:** Xiaohua Wang, Hongju Kou, Huiliao He, Mingdong Lu, Lingling Zhou, Liang Wang

**Affiliations:** ^1^Department of Ultrasound, The Second Affiliated Hospital and Yuying Children's Hospital of Wenzhou Medical University, Wenzhou, China; ^2^Department of Gastrointestinal Surgery, The Second Affiliated Hospital and Yuying Children's Hospital of Wenzhou Medical University, Wenzhou, China; ^3^Department of Pathology, The Second Affiliated Hospital and Yuying Children's Hospital of Wenzhou Medical University, Wenzhou, China

**Keywords:** gastric cancer, gastric stromal tumor, ultrasonography, perfusion parameter, differential diagnosis

## Abstract

**Objective:** To explore the difference of perfusion parameters between gastric cancer (GC) and gastric stromal tumors (GSTs) by using oral contrast plus contrast-enhanced ultrasonography (OC+CEUS).

**Methods:** We retrospectively reviewed 149 patients with histologically confirmed gastric lesions (80 patients with GC and 69 patients with GST). OC+CEUS was performed in all patients in the GC group and the GST group before surgery. The cine loops of OC+CEUS of all cases were analyzed. The perfusion parameters including arrival time (AT), time to peak (TTP), basal intensity (BI), and peak intensity (PI) were obtained via a program designed for autotracking contrast quantification (ACQ). The between-group differences in these parameters were compared.

**Results:** According to time-intensity curve (TIC) analysis, high-risk GST had higher PI than low-risk GST (*P* < 0.05). GC had faster AT and higher PI than normal gastric wall (*P* < 0.05); GST had higher PI than normal gastric wall (*P* < 0.05). Furthermore, the GC group had faster AT and higher PI than the GST group (*P* < 0.05). In contrast, the difference in BI and peak time (TTP) between the groups was not significant (*P* > 0.05).

**Conclusion:** AT and PI differ significantly between the GC group and the GST group. As a new method, OC+CEUS has value for the differential diagnosis of GC and GST.

## Introduction

Gastric cancer (GC) is a malignant tumor that originated from gastric epithelial tissue. It is the fourth most common cancer with ~1 million new cases annually and is the third leading cause of cancer-associated death globally (1, 2). The reasons for its associated high mortality include its innate aggressiveness and the fact that it is often only detected once it has become advanced, with ~60% of patients having disease that is locally or systemically advanced upon diagnosis (3). Despite recent development of diagnostic and therapeutic methods, the 5-years overall survival (OS) of the disease remains poor and ranges from 10 to 30% (4, 5). Gastric stromal tumor (GST) is a neoplasm that originated from gastric mesenchymal tissue with potential malignant features. It is a relatively rare tumor type, but can be readily cured in many cases, with patients having 5-years OS rates of 60–85% (6). Indeed, the prognosis for GST patients with advanced disease has improved significantly in recent years, whereas similar gains have not been made with respect to GC (3, 7). The orally administered tyrosine kinase inhibitor imatinib is one of the key mediators of these survival gains in GST patients (8, 9); the identification of novel c-KIT mutations and the combination of imatinib with other therapeutic agents have helped to further treat those affected by GST (10). Due to the large difference in the treatment and prognosis between GA and GST, it is necessary to differentiate GA from GST before therapeutic strategy is planned.

GC is a common tumor derived from the epithelium of the stomach. The early GC is confined to the mucosa and the submucosa. The advanced GC infiltrates below the submucosa. Unlike GC, GSTs originate from the muscularis propria. Small GSTs often form solid subserosal, intramural masses and in some cases are instead polypoid intraluminal masses. A majority of larger GSTs form external masses on the outer gust aspect, with muscular layer involvement, with larger such tumors often being cystic in the center. Some GSTs have an asymmetric hourglass-like pattern with a smaller internal and a larger external component. Many imaging modalities are used to identify gastric tumors, including barium studies, computed tomography, magnetic resonance imaging endoscopy, and endoscopic ultrasonography (EUS) (11, 12). There are, however, disadvantages to these approaches. For examine, MRI costs are high and can be contraindicated in patients with cochlear or pacemaker implants, while CT and barium examinations necessitate exposure to ionizing radiation. As GSTs grow typically in the muscularis and subserosa, when small they can be hard to recognize upon endoscopic examination; EUS is routinely used to detect gastrointestinal tumors and provides detailed images (13), but patients' discomfort and associated infection risk prevent its widespread use.

Oral contrast plus contrast-enhanced ultrasonography (OC+CEUS) combines intravenous microbubbles with oral contrast-enhanced ultrasonography, and was designed to serve as a novel mode of detecting stomach diseases such as GC in China (14, 15). However, no published study has compared the perfusion parameters of GC and GST by using OC+CEUS. We, therefore, conducted a retrospective cohort study comparing the perfusion parameters of these two diseases.

This study aimed to evaluate the differences in the perfusion parameters between the GC and GST groups, and to emphasize the practicability and usefulness of OC+CEUS in the clinical differential diagnosis of GC and GST.

## Methods

This study was carried out in accordance with the Declaration of Helsinki. The protocol was approved by the Research Ethics Committee of the Second Affiliated Hospital of Wenzhou Medical University. Informed consent was obtained from each patient.

### Patients

Between January 2012 and October 2019, 96 cases of GC and 75 cases of GST were enrolled into this study. The inclusion criteria for the study were as follows: ①Patients had undergone OC+CEUS examination within 1 week prior to surgery. ②The analyzed lesions were primary lesions. ③Patients had not received any prior cancer-related treatments. Exclusion criteria were as follows: ①OC+CEUS image quality was not sufficient to permit quantitative analyses; and ②patients with gastrointestinal surgical history. The final GC group consisted of 80 patients [27 females, 53 males, mean age 59.3 ± 9.2 years (range 32–83)]. The final GST group consisted of 69 patients [33 males, 36 females, mean age 53.6 ± 10.1 years (range 27–69)].

### Equipment and Reagents

An Acuson Sequoia 512 machine equipped with a 4V1 transducer and the microbubble-specific contrast pulse sequencing (CPS) technology was used for OC+CEUS assessment. The Xinzhang oral contrast agent (Huqingyutang, HangZhou, China), which is made of a soya derivative, was used in these ultrasonography studies, whereas SonoVue (Bracco, Italy) was intravenously injected for intravenous contrast and was composed of sulfur hexafluoride microbubbles.

### OC+CEUS Examinations

Patients were required to fast for a 6-h period before undergoing OC+CEUS examination, and at 30 min before injection, they were administered with 0.5 mg intramuscular atropine to reduce peristalsis during the imaging study. The stomach was first subjected to a baseline 2D ultrasonography scan in order to locate the lesions in the patient, with the machine being operated in the fundamental mode with a grayscale and a multifrequency 4V1 convex array probe. Following lesion localization, patients consumed 500 ml of an ultrasonic oral contrast agent (UOCA), after which they were assessed in the supine, left lateral, and right lateral positions during full inspiration. For each of these examinations, lesions in the stomach were examined, with their sizes, shapes, and echoic features being recorded. Patients were then administered with a bolus of Sonovue (2.4 ml, administered intravenously with a 19-Gauge cannula) followed by a flush with 3–5 ml saline. OC+CEUS was then conducted in contrast pulse sequencing (CPS) mode with the following settings: transmit frequency, 1.5 MHz; acoustic power, −15 to −21 dB; frame rate, 17–20. To reduce disruption associated with the microbubbles being used for contrast, we selected a low mechanical index (<0.2). Gastric lesion enhancement patterns were then digitally recorded for a 5-min period in order to capture the arterial, venous, and late phases.

**Figure 1:**
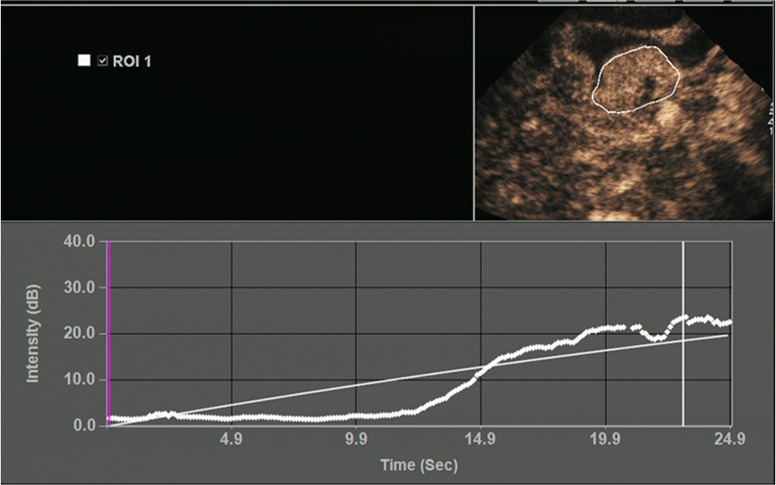
Time-intensity curve (TIC) image of gastric lesion. ROI, region of interest.

**Figure 2:**
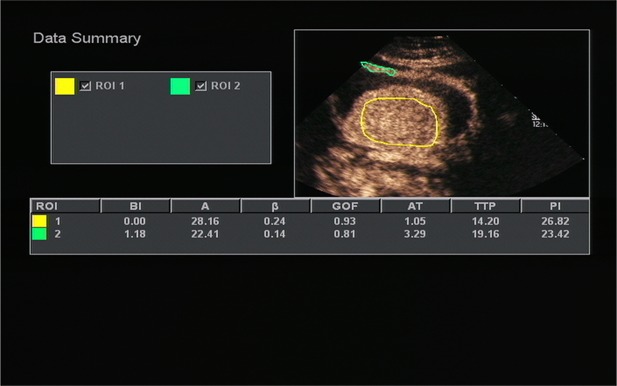
Acoustic quantitative analysis chart of a 59-years-old man with Borrmann type I gastric cancer. BI, basal intensity; GOF, goodness of fit; AT, arrival time; TTP, time to peak; PI, peak intensity. ROI 1 (yellow) indicated the ROI of the cancer; ROI 2 (green) indicated the ROI of the surrounding normal gastric wall.

**Figure 3:**
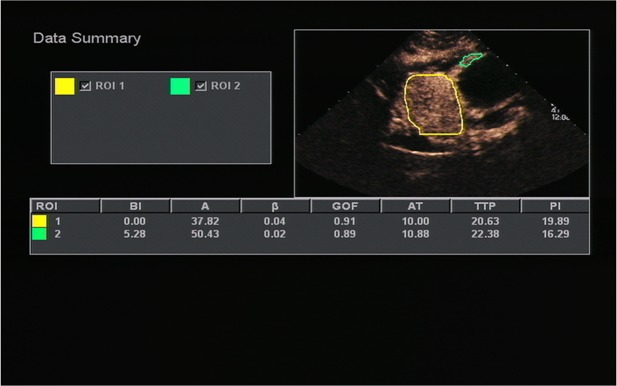
A 51-years-old man with gastric stromal tumor. Acoustic quantitative analysis chart showed the perfusion parameters of both stromal tumor (ROI 1) and surrounding normal gastric wall (ROI 2).

All OC+CEUS examinations were performed and recorded by a sonographer (HH). The cine loops were reviewed and analyzed by another sonographer (XW). Both of these professionals were blinded to patient clinical data, or pathology/imaging findings, and both had >10 years' experience. By using the quantitative analysis software, the region of interest (ROI) should envelop the lesion as a whole as possible to acquire the time-intensity curve (TIC) (Figure 1). The perfusion parameters of OC+CEUS including arrival time (AT), time to peak (TTP), basal intensity (BI), and peak intensity (PI) were obtained by the ACQ software (Figures 2, 3). Frame-by-frame manual adjustment of ROIs was performed to minimize breathing-related motion artifacts. Goodness of fit (GOF) should be >0.75.

### Statistical Analysis

SPSS v22.0 was used for all statistical testing. All OC+CEUS parameters were expressed as mean ± standard deviation (mean ± SD). The differences between the low-risk GST group and the high-risk GST group were compared by independent-sample *t*-tests. The differences between GC or GST and its normal gastric wall were compared by paired-sample *t*-tests. The differences between the GC group and the GST group were compared by independent-sample *T*-tests; *P* < 0.05 was considered statistically significant.

## Results

**Figure 4:**
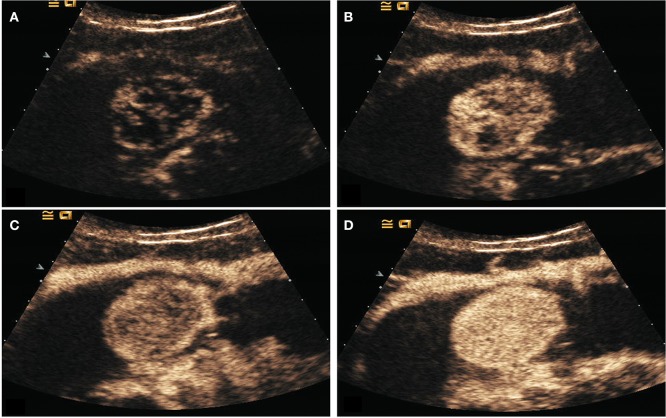
DECUS images of a 46-years-old woman with gastric stromal tumor. **(A–D)** showed the lesion was enhanced from the periphery to the center during the arterial phase and performed as a centripetal filling enhancement pattern.

All patients underwent surgery. Gastric lesions in the GC group had diameters that ranged from 1.3 to 8.9 cm (mean 4.6 ± 1.5 cm). The histological classifications were as follows: 22 cases of well-differentiated adenocarcinoma, 34 cases moderately differentiated adenocarcinoma, 21 cases of poorly differentiated adenocarcinoma, and 3 cases of signet-ring carcinoma. The diameters of the resected lesions in the GST group ranged from 1.5 to 13.9 cm (mean 5.7 ± 1.8 cm). The histological classifications were the low-risk group (33 cases), the moderate-risk group (8 cases), and the high-risk group (28 cases) (Table 1). On the oral contrast, the gastric cavity performed as a mid-gray, homogeneous region that acts as an acoustic window and improves the visualization of the inner wall. GCs appeared as thickened gastric wall or ulcerating hypoechoic lesions that protrude into the gastric cavity. After bolus injections of Sonovue, 45/80 lesions demonstrated homogeneous enhancement and 35/80 lesions demonstrated heterogeneous enhancement. On the oral contrast, GSTs appeared as round, oval, lobulated, or dumbbell-shaped hypoechoic masses with internal homogeneous or heterogeneous echotexture. After injection of intravenous contrast, 56/69 lesions enhanced from periphery to the center and had a peripheral ring-like hyper-enhancement sign (Figure 4); 41/69 lesions showed homogeneous enhancement, and 28/69 lesions showed heterogeneous enhancement.

**Table 1:** Patients characteristics of the gastric cancer group and the gastric stromal tumor group.

	**GC group**		**GST group**
Cases, *n*	80		69
Gender, male/female	53/27		33/36
Age, *y*, mean ± SD	59.3 ± 9.2		53.6 ± 10.1
Size, cm, mean ± SD	4.6 ± 1.5		5.7 ± 1.8
**Classification**, ***n***			
Adenocarcinoma			
Well-differentiated	22	Low risk	33
Moderately differentiated	34	Moderate risk	8
Poorly differentiated	21	High risk	28
Signet-ring carcinoma	3		

*GC, gastric cancer; GST, gastric stromal tumor*.

The mean AT, TTP, BI, and PI values of low-risk GST were 9.29 ± 2.42 s, 21.74 ± 5.23 s, 1.22 ± 0.71 dB, and 17.63 ± 3.90 dB, respectively. The mean values of AT, TTP, BI, and PI of high-risk GST were 8.65 ± 2.81 s, 21.18 ± 5.49 s, 1.34 ± 0.68 dB, and 19.51 ± 3.00 dB, respectively. There was no significant difference in AT, BI, and TTP between low-risk GST and high-risk GST (*P* > 0.05). The PI of the high-risk group was higher than that of the low-risk group (*P* < 0.05) (Table 2).

**Table 2:** Comparison of the perfusion parameters between the low-risk GST group and the high-risk GST group ($\bar{x}\pm$ s).

**Group**	***n***	**AT (s)**	**TTP (s)**	**BI (dB)**	**PI (dB)**
Low-risk GST	33	9.29 ± 2.42	21.74 ± 5.23	1.22 ± 0.71	17.63 ± 3.90
High-risk GST	28	8.65 ± 2.81	21.18 ± 5.49	1.34 ± 0.68	19.51 ± 3.00
*P*		*P* = 0.338	*P* = 0.336	*P* = 0.501	*P* = 0.042^*^

*GST, gastric stromal tumor; AT, arrival time; TTP, time to peak; BI, basal intensity; PI, peak intensity*.

* *P < 0.05*.

The mean AT, TTP, BI, and PI values of t GC group were 7.95 ± 2.21 s, 21.74 ± 5.23 s, 1.31 ± 0.73 dB, and 19.98 ± 3.28 dB, respectively. The mean AT, TTP, BI, and PI values of the normal gastric wall in GC patients were 9.30 ± 2.65 s, 22.46 ± 4.45 s, 1.39 ± 0.71 dB, and 17.75 ± 2.90 dB, respectively. There was no significant difference in BI and TTP between GC and the normal gastric wall (*P* > 0.05). GC had faster AT and higher PI than the normal gastric wall (*P* < 0.05) (Table 3). The mean values of AT, TTP, BI, and PI of the GST group were 9.14 ± 2.54 s, 21.18 ± 5.49 s, 1.27 ± 0.67 dB, and 18.41 ± 3.45 dB, respectively. The mean AT, TTP, BI, and PI values of the normal gastric wall in GST patients were 9.42 ± 2.57 s, 21.66 ± 5.07 s, 1.40 ± 0.76 dB, and 17.76 ± 3.17 dB, respectively. There was no significant difference in AT, BI, and TTP between GST and the normal gastric wall (*P* > 0.05). GST had higher PI than the normal gastric wall (*P* < 0.05) (Table 4). There was no significant between-group difference in BI and TTP (*P* > 0.05). The AT of the GC group was faster than that of the GST group, and the PI of the GC group was higher than that of the GST group (*P* < 0.05) (Table 5).

**Table 3:** Comparison of the perfusion parameters between GC and the normal gastric wall ($\bar{x}\pm$ s).

**Group**	**AT (s)**	**TTP (s)**	**BI (dB)**	**PI (dB)**
GC	7.95 ± 2.21	21.74 ± 5.23	1.31 ± 0.73	19.98 ± 3.28
Normal gastric wall	9.30 ± 2.65	22.46 ± 4.45	1.39 ± 0.71	17.75 ± 2.90
*P*	*P* = 0.002^*^	*P* = 0.137	*P* = 0.535	*P* = 0.000^*^

*GC, gastric cancer; AT, arrival time; TTP, time to peak; BI, basal intensity; PI, peak intensity*.

* *P < 0.05*.

**Table 4:** Comparison of the perfusion parameters between GST and the normal gastric wall ($\bar{x}\pm$ s).

**Group**	**AT (s)**	**TTP (s)**	**BI (dB)**	**PI (dB)**
GST	9.14 ± 2.54	21.18 ± 5.49	1.27 ± 0.67	18.41 ± 3.45
Normal gastric wall	9.42 ± 2.57	21.66 ± 5.07	1.40 ± 0.76	17.76 ± 3.17
*P*	*P* = 0.078	*P* = 0.138	*P* = 0.098	*P* = 0.003^*^

*GST, gastric stromal tumor; AT, arrival time; TTP, time to peak; BI, basal intensity; PI, peak intensity*.

* *P < 0.05*.

**Table 5:** Comparison of the perfusion parameters between the two groups ($\bar{x}\;\pm$ s).

**Group**	**AT (s)**	**TTP (s)**	**BI (dB)**	**PI (dB)**
GC	7.95 ± 2.21	21.74 ± 5.23	1.31 ± 0.73	19.98 ± 3.28
GST	9.14 ± 2.54	21.18 ± 5.49	1.27 ± 0.67	18.41 ± 3.45
*P*	*P* = 0.003^*^	*P* = 0.518	*P* = 0.705	*P* = 0.005^*^

*GC, gastric cancer; GST, gastric stromal tumor; AT, arrival time; TTP, time to peak; BI, basal intensity; PI, peak intensity*.

* *P < 0.05*.

## Discussion

OC+CEUS is a transabdominal ultrasound approach combining intraluminal and intravenous contrast agents to enhance sonographic imaging results (16). The filling of the stomach with UOCA leads to the elimination of any gas therein, producing a uniform interface that allows for more effective ultrasonic transmission, thus significantly reducing ultrasonic artifact formation and allowing the gastric wall to display more clearly, enabling stomach lesions to be more readily detected (17). Invasive tumors typically exhibit angiogenesis and infiltration (18). Single usage of an oral contrast agent is unable to display the microvascular perfusion of the lesions. SonoVue is a pure blood pool contrast agent, which can produce strong echogenicity over the range of frequencies used in medical ultrasound examinations (19). Intravenous injection of SonoVue can enter the microvessels of gastric tumors through blood circulation. It can display blood perfusion of the tumors and enhance visualization of lesions during the arterial phase to identify the boundaries of invasion. In addition, the TIC and perfusion parameters obtained by using the ACQ software after injection of SonoVue can be used to quantitatively evaluate the microcirculation perfusion of tumor tissues (20). Thus, OC+CEUS is able to demonstrate both morphologic appearances and perfusion status of gastric lesions (15).

**Figure 5:**
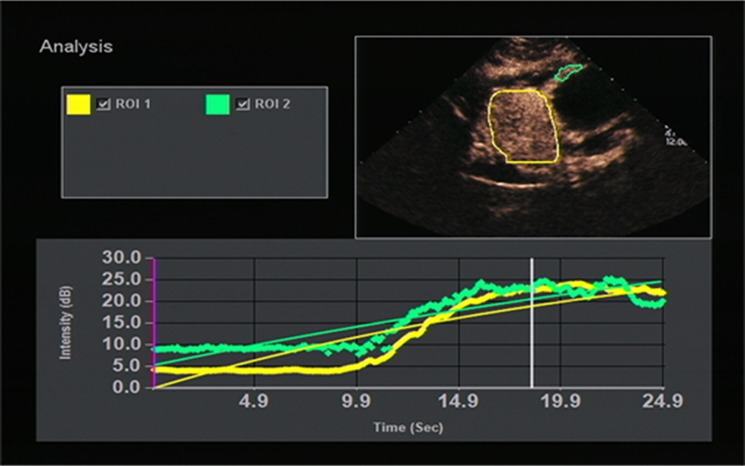
TIC image showed the stromal tumor (yellow curve) enhanced almost synchronously with the surrounding normal gastric wall (green curve).

The results of this study showed that high-risk GST had higher PI than low-risk GST (*P* < 0.05); GC had faster AT and higher PI than the normal gastric wall (*P* < 0.05); GST had higher PI than the normal gastric wall (*P* < 0.05); and AT in the GC group was faster than that in the GST group, and PI in the GC group was higher than that in the GST group according to quantitative analysis (*P* < 0.05). AT indicates the time interval during which the intensity of the contrast agent in targeted tissues changes significantly from the initial state. The AT of GC was faster than that of the normal gastric wall and GST, which was correlated with the presence of arteriovenous shunts and a well-represented circulatory bed of GC tissues (21). And that led to the rapid wash-in of the contrast agent in the GC group. Unlike GC, most GSTs locate in the muscularis and the layers of gastric walls are not destroyed. The blood flow from both mucosa and serosa supplies GSTs in the muscular layer, forming a blood flow perfusion pattern from the periphery to the center (Figure 4). So, most lesions in the GST group enhanced almost synchronously with the surrounding normal gastric wall (Figure 5) and performed as a centripetal filling enhancement pattern. That is why the AT of GST was slower than that of GC, and there was no significant difference in AT between GST and the normal gastric wall. PI is correlated with the maximum dose of the contrast agent that reaches the lesion, and it is proportional to the average blood flow volume in the ROI. It has been reported that the expression level of the vascular endothelial growth factor in the high-risk GST is higher than that in the low-risk GST (22). An important function of the vascular endothelial growth factor is the formation of new blood vessels, which indicates that the high-risk group has more new blood vessels. Therefore, the PI of the high-risk GST group is higher than that of the low-risk GST group. GST has more abundant vascular network than GC (23); the corresponding PI should be higher than GC. But in our study, the PI of the GC group was higher than that of the GST group. GST is prone to hemorrhage and necrosis, which leads to its reduced internal blood flow, so the concentration of the internal contrast agent decreased. That is why the PI of GST was lower than that of GC.

This study was a retrospective study of only patients that had been referred for surgical treatment. This issue represents a bias that could affect the accurate evaluation. We think we should do a prospective study to avoid bias in our future research.

In conclusion, there is a significant difference in AT and PI between GC and GST. As a relatively new, convenient, and non-invasive method, OC+CEUS is valuable in the differential diagnosis of GC and GST. It could be a useful tool before therapeutic strategies is planned.

## Data Availability Statement

All datasets generated for this study are included in the article/supplementary material.

## Ethics Statement

The studies involving human participants were reviewed and approved by The Research Ethics Committee of the Second Affiliated Hospital of Wenzhou Medical University. The patients/participants provided their written informed consent to participate in this study. Written informed consent was obtained from the individual(s) for the publication of any potentially identifiable images or data included in this article.

## Author Contributions

XW and LW designed this study and wrote and edited the manuscript. XW, HK, and ML acquired the data. HH and LZ interpreted the data. All authors reviewed the manuscript.

## Conflict of Interest

The authors declare that the research was conducted in the absence of any commercial or financial relationships that could be construed as a potential conflict of interest.
